# Culture moderates the relationship between interdependence and face recognition

**DOI:** 10.3389/fpsyg.2015.01620

**Published:** 2015-10-27

**Authors:** Andy H. Ng, Jennifer R. Steele, Joni Y. Sasaki, Yumiko Sakamoto, Amanda Williams

**Affiliations:** Department of Psychology, York University, Toronto, ON, Canada

**Keywords:** individual differences, cross-cultural differences, face recognition, interdependent self-construal, East Asian

## Abstract

Recent theory suggests that face recognition accuracy is affected by people’s motivations, with people being particularly motivated to remember ingroup versus outgroup faces. In the current research we suggest that those higher in interdependence should have a greater motivation to remember ingroup faces, but this should depend on how ingroups are defined. To examine this possibility, we used a joint individual difference and cultural approach to test (a) whether individual differences in interdependence would predict face recognition accuracy, and (b) whether this effect would be moderated by culture. In Study 1 European Canadians higher in interdependence demonstrated greater recognition for same-race (White), but not cross-race (East Asian) faces. In Study 2 we found that culture moderated this effect. Interdependence again predicted greater recognition for same-race (White), but not cross-race (East Asian) faces among European Canadians; however, interdependence predicted worse recognition for both same-race (East Asian) and cross-race (White) faces among first-generation East Asians. The results provide insight into the role of motivation in face perception as well as cultural differences in the conception of ingroups.

## Introduction

Humans are social beings who are motivated to form interpersonal relationships, maintain these relationships, and be included in social groups (Guisinger and Blatt, 1994; Baumeister and Leary, 1995; Brewer and Caporael, 2006). In order to engage successfully in social interactions and to coordinate activities with other group members, accurate face recognition is a basic and important requirement (Yardley et al., 2008). Given our limited cognitive capacity, however, we may only identify others as individuals when we have the motivation to do so (Fiske et al., 1999). Yet, what motivates one perceiver to individuate another may depend on both the individual and the larger cultural context. Building on this possibility, the main goal of the current research was to investigate whether people’s memory for novel faces could be predicted by their chronic motivations to make social connections using a culture × individual difference approach.

### Face Recognition as a Motivated Process

Research suggests that social motivation plays an important role in the way that faces are processed and subsequently remembered (DeWall and Maner, 2008; Hugenberg et al., 2010; Kawakami et al., 2014). It has been demonstrated that people have better memory for novel targets who belong to the same racial group, compared with those who belong to a different racial group (see Meissner and Brigham, 2001, for a review), an effect which has been termed the cross-race effect^1^. Although perceptual experience with members of different racial groups can contribute to the cross-race effect (e.g., Goldstein and Chance, 1985), there is recent evidence to suggest that the cross-race effect may also have a motivational component. For example, it has been found that when a common social group (i.e., university affiliation) is made salient, European American perceivers show the same face recognition accuracy for White and Black targets, eliminating the cross-race effect (Hehman et al., 2010). This suggests that people may be more *motivated* to remember the face of a target who is construed as an ingroup as opposed to an outgroup member.

### Individual Differences in Interdependence

People can vary substantially, however, in the extent to which they see themselves as being fundamentally connected to and interdependent with others in their social ingroups (Markus and Kitayama, 1991). Individuals who construe themselves as high in interdependence tend to value their connections with others in their ingroups, and their self-concepts are defined by relationships with ingroup members (Markus and Kitayama, 1991). Although interdependence was a psychological construct developed primarily in the context of cultural comparisons between East Asians and European North Americans, there is considerable variation among individuals within each culture in terms of their interdependent orientation (Triandis et al., 1985; Yamaguchi, 1994). Hence, interdependence has also been used as an individual difference variable, predicting social behaviors of various kinds (e.g., Holland et al., 2004; Schouten, 2007; Tams, 2008). We contend that chronically interdependent people may be particularly likely to show enhanced face memory for targets who are perceived to belong to an ingroup. However, research also suggests that how people define their ingroups can differ across cultures.

### Cultural Differences in Group Processes

Although the basic need to belong to social groups and connect with ingroup members is universal (Baumeister and Leary, 1995), research suggests that how people define their ingroups can differ cross-culturally. In North America, feeling a connection to someone who went to the same university as you, even if you have never met, may not seem out of the ordinary. Those at the same university might feel like ingroup members. Yet in some other cultures, such as in East Asia, this feeling may not be as prevalent. According to Brewer and Yuki (2007), North Americans rely heavily on abstract, categorical group memberships, including race, nationality, and university affiliation, when constructing social identities. By contrast, people in East Asian cultures often strive to maintain harmony and promote cohesion within ingroups that are more tightly defined, with members holding direct or indirect personal connections with each other (Brewer and Yuki, 2007). Bond (1991) also noted the exclusivity of the ingroup in some East Asian cultures, having observed a tendency to “make a critical distinction between established acquaintances and others” (p. 51).

This distinction seems to have implications for how people relate to strangers in their environment. Indeed, intergroup biases based on abstract social categorical distinctions tend to be less pronounced among East Asians compared with North Americans. For example, Japanese students are less likely to exhibit ingroup biases when evaluating others who belong to their own universities compared to North American students, who tend to show strong ingroup biases (Snibbe et al., 2003). Likewise, ingroup bias in trust toward people of the same university or of the same town of residence is weaker among Japanese participants compared with American participants (Yuki et al., 2005).

Due to differences in the conceptualization of what constitutes an ingroup between European North Americans and East Asians (Brewer and Yuki, 2007), we propose that the relationship between interdependence and novel same-race face recognition might depend on one’s cultural background. Whereas novel members of the same social category or group are known to elicit motivational biases among North Americans (Falk et al., 2014), these same motivational biases may not occur for East Asians insofar as there is no pre-existing relationship between them. Thus, we propose that for European Canadians, being higher in interdependence should increase one’s motivation to attend to and process ingroup faces, such as novel same-race faces, due to the heightened value that individuals high in interdependence place on connections with others in their ingroup, resulting in more accurate memory for these faces (see Van Bavel et al., 2012, for a related argument). For East Asians, we would not anticipate a positive relationship between interdependence and same-race face recognition to emerge as strangers are unlikely to be considered ingroup members, regardless of racial category.

### The Present Research

In the present research, we examined face recognition processes from a culture × individual difference perspective across two experiments using a standard face recognition paradigm. In Study 1 we tested European Canadian participants and hypothesized that individual differences in interdependence would be positively associated with same-race, but not cross-race, face recognition accuracy. In Study 2 we tested both European Canadian and first-generation East Asian Canadian participants, and hypothesized that culture would moderate the relationship between interdependence and same-race face recognition, such that interdependence would positively predict same-race face recognition accuracy for European Canadians, but not for East Asians.

## Study 1

### Method

#### Participants

The present study protocol was reviewed and approved by the Research Ethics Committee of York University, conforming to the Tri-Council Policy Statement on Ethical Conduct for Research Involving Humans. Twenty-four Canadian-born university students of European cultural backgrounds (11 female, *M*_age_ = 19.7 years) completed the present study for course credit.

#### Materials

***Face stimuli***

Forty-eight grey-scaled face photos were used in this study (Ekman and Matsumoto, 1993; Goldinger et al., 2009). These included 24 White (12 male) and 24 East Asian (12 male) faces with neutral expressions. The size of each face photo was 11.75 × 16 inches.

***Interdependence***

Interdependence was measured using the 12-item Interdependence subscale (α = 0.53)^2^ of the Self-Construal Scale (SCS; Singelis, 1994). Sample items include: “It is important for me to maintain harmony within my group” and “I will sacrifice my self-interest for the benefit of the group I am in,” rated on a 7-point scale, from strongly disagree to strongly agree.

#### Procedure

Seven to 14 days prior to completing the recognition task, participants were asked to complete an online survey. Participants first read an informed consent form and indicated their consent by checking a box on the form. Following this, they completed the SCS-Interdependence and other demographic questions (e.g., age, gender). The main study was conducted in a social psychology laboratory. Participants first read an informed consent form on a computer screen and indicated their consent by pressing a specified key. Then, participants were told that they would be shown faces that they should pay close attention to, as their memory for the faces would be subsequently tested. Each participant sat in front of a computer monitor as 24 pictures (12 White faces, 12 East Asian faces) were individually presented in the center of the monitor in a random order. Each photo was displayed for 10 s with an interstimulus interval of 2 s.

After a 5-min puzzle game, which served as a filler task, they completed the self-paced face recognition task. Participants viewed all of the 24 previously seen faces and 24 (12 White, 12 East Asian) new faces, presented individually and in a random order. Participants indicated whether they had seen each face previously by pressing one of two computer keys.

### Results and Discussion

We first computed face recognition accuracy scores using the signal detection parameter sensitivity (*d*′; Green and Swets, 1966) where *d*′ = z(hit) – z(false alarms) for White and East Asian faces separately.

To test our focal hypothesis, we examined the zero-order correlation between interdependence and face recognition accuracy for same-race (White) faces. As expected, this correlation was significant, *r*(24) = 0.41, *p* < 0.05, with European Canadians higher in interdependence showing better memory for novel White faces. In line with our expectation that this finding would not extend to cross-race faces, interdependence and participants’ memory for cross-race (East Asian) faces were not related, *r*(24) = –0.04, *p* = 0.85. The difference between these two correlations was statistically significant, *z* = 2.00, *p* < 0.05, Cohen’s *q* = 0.21.

These results support our hypothesis that higher levels of interdependence predict enhanced memory for same-race, but not cross-race, faces among European Canadian perceivers. These findings are consistent with our expectation that European Canadians who place greater value on connections with other ingroup members, as reflected in higher self-reported interdependence, show increased motivation to process same-race faces, leading to better memory for these faces.

## Study 2

In Study 2, we first sought to replicate the effect observed in Study 1. Using a larger sample and a different set of face stimuli, we again examined whether, for European Canadians, interdependence, as an individual difference variable, would be positively associated with face recognition accuracy for same-race faces. In addition, we extended the results of Study 1 by testing our culture × individual difference hypothesis. As East Asians tend to define their ingroup based on close social relationships rather than shared social categories (Brewer and Yuki, 2007) and generally show less orientation toward and biases favoring unfamiliar others, even when they share a broader social category (Bond, 1991; Snibbe et al., 2003), we anticipated a moderating effect of culture on the positive relationship between interdependence and same-race face recognition. Specifically, we hypothesized that a positive relationship between interdependence and same-race face recognition would be observed among European Canadian, but not East Asian, perceivers.

### Method

#### Participants

The present study protocol was reviewed and approved by the Research Ethics Committee of York University, conforming to the Tri-Council Policy Statement on Ethical Conduct for Research Involving Humans. One hundred and twenty-seven undergraduates, including 67 (49 female; *M*_*age*_ = 22.4 years) self-identified European Canadians and 60 (43 female; *M*_*age*_ = 22.0 years) self-identified first-generation East Asians living in Canada completed the present study for course credit. Forty-eight of the European Canadians were born in Canada and 19 were born in the United States or a European country (e.g., Germany). For those who were not born in Canada, the average length of residence in Canada was 10.0 years (SD = 7.53). All of the first-generation East Asians were born in an East Asian country (e.g., China, Taiwan, Korea) with the average length of residence in Canada being 10.1 years (SD = 5.02).

#### Materials

***Face stimuli***

One hundred and eighty gray-scaled face photos were used in this study, including 60 White, 60 East Asian, and 60 Black male targets each displaying a neutral facial expression^3^. The size of each face photo was 6 × 5.25 inches.

***Interdependence***

The same 12-item Interdependence subscale (α = 0.69 for European Canadians; α = 0.77 for East Asians) of the SCS (Singelis, 1994) was used as in Study 1^4^.

#### Procedure

Participants first read an informed consent form on a computer screen and indicated their consent by pressing a specified key. Following this, participants completed the face learning phase in which they viewed 90 faces (30 White, 30 East Asian, 30 Black) presented individually for 3 s (interstimulus interval of 0.5 s) at the center of a computer monitor in random order. Each participant was asked to pay attention to the faces as they would subsequently perform a memory test. After completing some unrelated filler tasks for 8–10 min, participants completed the self-paced face recognition task. Each participant viewed 90 faces that had been previously seen in the learning phase and 90 (30 White, 30 East Asian, 30 Black) new faces, presented individually and in a random order, in the middle of the computer screen. As in Study 1, participants pressed one of two keys to indicate whether they had seen each face previously, and each face remained until a response was made. Finally, participants completed the SCS-Interdependence as well as some demographic questions (e.g., age, gender), and were debriefed.

### Results and Discussion

As in Study 1, we first computed face recognition accuracy scores using *d*′ (Green and Swets, 1966; see Study 1). In order to test our prediction that interdependence would relate to face recognition accuracy differently as a function of perceiver’s cultural background and target race, we conducted a series of regression analyses. In each analysis, culture (European Canadian = 0, East Asian = 1), interdependence (grand-mean-centered), and the interaction term of culture and interdependence were entered simultaneously to examine the moderating effect of culture. Face recognition accuracy (*d*′) was used as the criterion.

**FIGURE 1:**
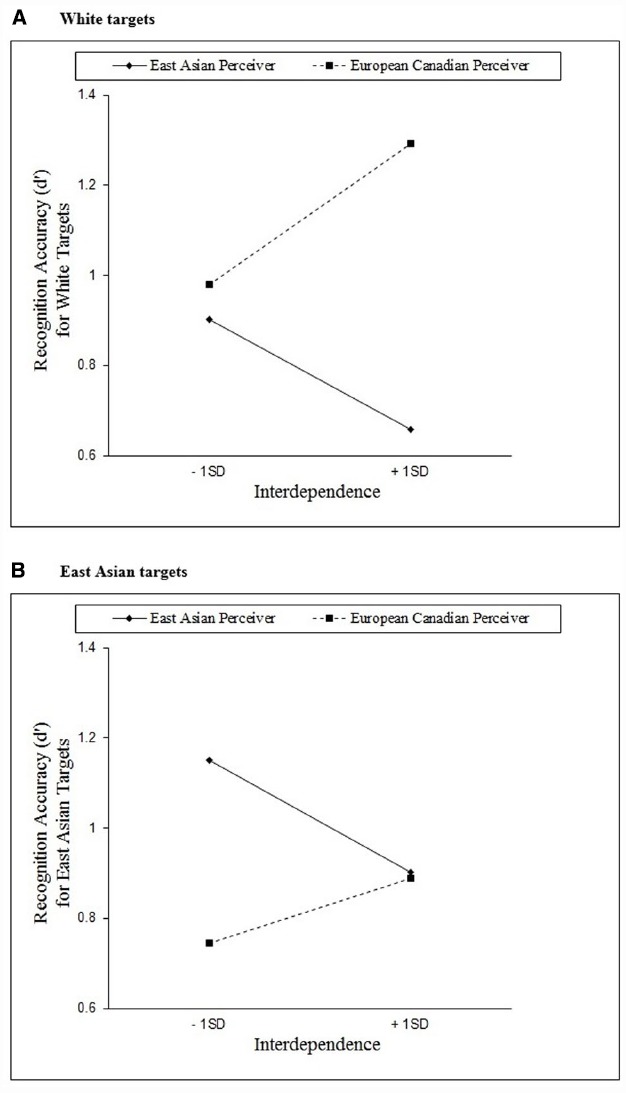
**The relationship between interdependence and face recognition for (A) White targets and (B) East Asian targets for first-generation East Asian and European Canadian perceivers in Study 2**.

In terms of recognition accuracy for White faces, the overall model was significant, *F*(3,120) = 8.25, *p* < 0.001, *R*^2^ = 0.17. Importantly, the predicted interaction effect emerged, β = 0.38, *p* < 0.01. Simple slope analyses (see Figure 1A) revealed a positive relationship between interdependence and face recognition accuracy for White (same-race) faces among European Canadian perceivers, *b* = 0.20, *p* = 0.03, consistent with the results of Study 1. By contrast, there was a negative relationship between interdependence and face recognition accuracy for White (cross-race) faces among East Asian perceivers, *b* = –0.15, *p* = 0.04.

In terms of recognition accuracy for East Asian faces, the overall model was again significant, *F*(3,120) = 5.00, *p* < 0.01, *R*^2^ = 0.11, and a significant interaction effect emerged, β = –0.34, *p* = 0.01. Consistent with the results of Study 1, simple slope analyses (see Figure 1B) indicated that there was no relationship between interdependence and face recognition accuracy for East Asian (cross-race) faces among European Canadian perceivers, *b* = 0.09, *p* = 0.20. On the other hand, among East Asian perceivers, a negative relationship between interdependence and face recognition accuracy emerged for East Asian (same-race) faces, *b* = –0.16, *p* = 0.02; greater interdependence was associated with *worse* memory for East Asian faces among East Asian participants^5^.

These results once again suggest that for European Canadians, higher levels of interdependence are associated with enhanced recognition for faces belonging to the same racial category, but not for faces belonging to a different racial category. By contrast, for East Asians, interdependence is associated with *decreased* recognition accuracy for novel faces, regardless of their racial category.

## General Discussion

As inherently social beings, humans have been living in groups across history. Building on recent research that has examined the role of social motivation in face processing, in the present research we examined whether individual differences in the self-reported value that people place on social connections with others in their ingroups is predictive of memory for novel faces. Through two studies we have demonstrated that face recognition accuracy can be predicted by individual differences in interdependence and that the direction of this relationship is moderated by one’s cultural background.

The results from Study 1 are consistent with current theorizing on the motivated nature of group-based face recognition biases (Hugenberg et al., 2010) and contribute to this literature by providing evidence that chronic interdependent orientation is positively related to face memory for novel same-race, but not cross-race, targets. In Study 2, we replicated this finding and extended these results by showing the role that culture plays in moderating the relationship between interdependence and face recognition biases. Consistent with theorizing on how group representation and related processes differ across cultures (Yuki, 2003; Brewer and Yuki, 2007), we did not find a positive relationship between interdependence and recognition accuracy for novel same-race faces among East Asian participants; instead, we found a negative relationship for both East Asian (same-race) and White (cross-race) faces. We speculate that this negative relationship between interdependence and novel face recognition may reflect the possibility that highly interdependent East Asians are those who put a high premium on maintaining meaningful connections and social relationships with close ingroup members (e.g., family members, friends), and thus these individuals may actually be less motivated to attend to people who are outside of their tightly knit web of social connections. This possibility is consistent with the recent findings that activating the concept of interdependence decreases empathic neural responses to a stranger (Jiang et al., 2014) but increases empathic neural responses to a friend (Varnum et al., 2014) among the Chinese, highlighting the tight and exclusive nature of their ingroups.

One limitation of the present research is that we did not examine the mediating mechanism. We expect that for European Canadians who are higher in interdependence, enhanced face recognition accuracy of racial ingroup members is driven by increased attention allocated to the individuating features of the face as well as the total attentional resources spent on processing those faces. In future research, it would be useful to examine attention when people process novel faces using eye-tracking technology (e.g., Goldinger et al., 2009; Kawakami et al., 2014) to gain better insight into this potential mediator. A second limitation is that East Asian immigrants in Canada were tested rather than East Asian nationals. However, due to the bicultural status of East Asian immigrants, who have likely adopted aspects of mainstream European Canadian cultural values to varying degrees, we would expect our findings to be at least as pronounced among East Asian nationals. Through future research it would be interesting to not only replicate these findings with East Asian nationals but to extend this research by examining second- and third-generation East Asian Canadians, who are more likely to have adopted mainstream European Canadian cultural values (e.g., the importance of positive self-regard, Heine et al., 1999) and might therefore show a positive relationship between interdependence and memory for same-race faces.

Building on these findings, it would also be useful for future research to investigate whether culture moderates the effect of other social motivations on face recognition accuracy. For example, researchers in North America have found that targets of relatively high social status capture more visual attention and are more likely to be remembered than those who are of relatively low social status (DeWall and Maner, 2008; Ratcliff et al., 2011). It seems likely that this effect would be more pronounced in vertical cultures, such as India (Triandis, 1995), where social hierarchies tend to be very rigid and the social interactions of people in these cultural contexts tend to be highly influenced by social status.

Finally, the current results have more general implications for cultural psychology. There is evidence to suggest that, in addition to the degree of collectivism, the nature of collectivism may also be qualitatively different across cultures (e.g., Triandis, 1995; Brewer and Yuki, 2007). Similarly high levels of interdependent social orientation at the individual level may have divergent, rather than similar, psychological consequences in different sociocultural contexts. Hence, it is crucial to take into consideration both cultural and individual level variables, and in particular how these may interact, when predicting human cognitions and behaviors (see Leung and Cohen, 2011). The current results attest to this possibility by showing that individual differences can have opposing relationships with among participants of European and East Asian cultural backgrounds.

### Conflict of Interest Statement

The authors declare that the research was conducted in the absence of any commercial or financial relationships that could be construed as a potential conflict of interest.

## Footnotes

1 In line with this literature (e.g., MacLin and Malpass, 2001), we use the term “race” to refer to a socially constructed way of categorizing people based on perceived physical appearance.

2 Three items were removed because their corrected item-total correlations were negative in this sample; however the pattern of results remained consistent with our hypothesis when the full scale was retained. The correlation between interdependence and face recognition accuracy was significant for same-race (White) faces (*p* < 0.05) but not cross-race (East Asian) faces (*p* = 0.50).

3 The White and Black face stimuli were adapted from Minear and Park (2004) and Blair et al. (2002). The East Asian face stimuli were adapted from the CAS-PEAL-R1 face database (Gao et al., 2008). In order to examine the generalizability of the results of Study 1, we increased the difficulty of the face recognition task by increasing the number of photos, decreasing the presentation time, and increasing the interval between the face-learning phase and the face-recognition phase in Study 2.

4 We retained all 12 items of the Interdependence subscale of the SCS in Study 2. For the sake of completeness we did administer the full SCS (which includes Independence and Interdependence subscales) in Studies 1 and 2, despite the fact that we had no *a priori* hypotheses regarding how independence might relate to face memory. However, we examined these relationships *post hoc*, at the suggestion of one of the reviewers. In Study 1, independence was positively correlated with face recognition for both White and East Asian targets, *p*s < 0.04. In Study 2, for European Canadian participants, independence was not correlated with face recognition for targets of all races, *p*s > 0.31. For East Asian participants, independence was negatively correlated with face recognition for White and Black targets, *p*s < 0.03, but not East Asian targets, *p* > 0.05.

5 In terms of face recognition accuracy for Black targets, the overall model was not significant, *F*(3,120) = 1.22, *p* = 0.31 and the interaction effect did not reach conventional level of significance, β = –0.20, *p* = 0.15. Although this interaction fell short of statistical significance, we performed simple slope analyses to further explore the results. We found that there was no relationship between interdependence and face recognition accuracy for Black (cross-race) faces among European Canadian perceivers, *b* = 0.10, *p* = 0.24, nor among first-generation East Asian perceivers, *b* = –0.07, *p* = 0.41. It is interesting that the negative relationship between interdependence and face recognition among East Asian perceivers did not extend to Black (cross-race) targets. Considering that face recognition is known to be jointly influenced by both contact experience and motivation (Young and Hugenberg, 2012) and the effect of motivation on cross-race face recognition seems to work only for perceivers who have relatively high level of contact experience with people of the other race (Young and Hugenberg, 2012, Study 1), one possible reason for this non-significant finding is that our East Asian immigrant participants might not have much contact experience with Black Canadians.
